# Retraction: DA Negatively Regulates IGF-I Actions Implicated in Cognitive Function via Interaction of PSD95 and nNOS in Minimal Hepatic Encephalopathy

**DOI:** 10.3389/fncel.2018.00349

**Published:** 2018-09-24

**Authors:** Frontiers Editorial Office

The journal retracts the 6 September 2017 article cited above. Following publication, the publisher was alerted to potential instances of image manipulation. In accordance with our established procedures, an investigation was initiated. The investigation revealed image duplication in Figure 8 and copy-paste marks and image manipulation in western blot gels (Figures 6, 7, 10). The authors and their institution have remained unresponsive to multiple requests for the raw data for this study. Given the authors' unresponsiveness and extant concerns over the validity of the study, the article has been retracted.

The retraction of the article was approved by the Chief Editors of Cellular Neuroscience and the Editor-in-Chief of Frontiers.

